# From Promise to Practice: Harmonizing Telemedicine in Pediatric Chronic Respiratory Diseases

**DOI:** 10.3390/jcm15041540

**Published:** 2026-02-15

**Authors:** Susanna Esposito, Daniele Donà, Giulia Brigadoi, Beatrice Rita Campana

**Affiliations:** 1 Pediatric Clinic, Department of Medicine and Surgery, University Hospital of Parma, 43126 Parma, Italy; beatricerita.campana@unipr.it; 2 Division of Pediatric Infectious Diseases, Department of Women’s and Children’s Health, University of Padua, 35128 Padua, Italy; daniele.dona@unipd.it (D.D.); giulia.brigadoi@unipd.it (G.B.)

**Keywords:** telemedicine, pediatrics, chronic respiratory diseases, core outcome set, health equity, implementation science

## Abstract

Telemedicine has the potential to substantially improve the care of children and adolescents with chronic respiratory diseases, including asthma, cystic fibrosis, bronchiectasis, and chronic respiratory failure. Digital health interventions—such as remote monitoring, virtual consultations, adherence-support tools, and educational platforms—can enhance disease control, continuity of care, and access to specialized services. Despite these opportunities, the implementation of telemedicine in pediatric respiratory care remains fragmented and uneven across healthcare systems. A central barrier to progress is the marked heterogeneity of outcome measures used to evaluate telemedicine interventions. Inconsistent definitions, variable endpoints, and limited follow-up reduce comparability across studies, hinder evidence synthesis, and impede translation into clinical guidelines, reimbursement models, and policy decisions. Consequently, telemedicine is often confined to isolated pilot projects rather than embedded within standard care pathways. This narrative review issues a Call to Action for the coordinated implementation and harmonization of telemedicine in pediatric chronic respiratory diseases. We advocate for the urgent development and adoption of a Core Outcome Set (COS) to standardize outcome measurement across clinical trials and real-world evaluations. In addition, we highlight the importance of integrating implementation science, economic evaluation, ethical oversight, and equity considerations into telemedicine research and deployment. Addressing regulatory fragmentation, ensuring interoperability, and aligning accreditation with reimbursement and Health Technology Assessment requirements are essential for sustainable scale-up. Finally, we emphasize the need for international collaboration among clinicians, researchers, policymakers, payers, technology developers, and patient advocacy groups to accelerate learning and promote equitable, evidence-based digital care models. Through coordinated action, telemedicine can evolve from a promising innovation into a reliable and accessible standard of care for children with chronic respiratory diseases.

## 1. Introduction

Chronic respiratory diseases in childhood, including asthma, cystic fibrosis, bronchiectasis, and chronic respiratory failure, represent a major cause of long-term morbidity worldwide [1,2,3]. These conditions have a profound impact on the quality of life of affected children and adolescents and impose a substantial emotional, social, and economic burden on families and healthcare systems. Their management requires continuous, multidisciplinary, and patient-centered care, characterized by regular monitoring, sustained treatment adherence, and effective coordination across primary, secondary, and tertiary care settings.

Over the past decade, and particularly following the acceleration driven by the COVID-19 pandemic, telemedicine and digital health technologies have emerged as promising tools to support the care of children with chronic respiratory diseases [4,5,6,7,8,9,10,11,12]. Remote monitoring, virtual consultations, symptom-tracking applications, adherence-support systems, and digital educational platforms offer new opportunities to improve access to care, enhance continuity, personalize treatment strategies, and facilitate timely clinical interventions. These approaches are especially relevant for pediatric populations, in whom frequent hospital visits can disrupt schooling, family life, and psychosocial well-being, and for families living in geographically remote or socioeconomically disadvantaged areas.

Despite this growing interest and technological availability, the real-world implementation of telemedicine in pediatric respiratory care remains inconsistent and fragmented. The existing evidence base is characterized by methodological limitations, including small sample sizes, short follow-up periods, and substantial heterogeneity in study designs and outcome measures. Most importantly, the lack of standardized outcomes undermines comparability across studies, limits the feasibility of robust meta-analyses, and hampers the translation of research findings into clinical guidelines, reimbursement policies, and scalable care models. Additional challenges persist in relation to regulatory frameworks, certification of digital tools, cost-effectiveness evaluation, data protection, and equity of access.

In this context, the aim of this paper is to serve as a Call to Action for the coordinated implementation and harmonization of telemedicine in pediatric chronic respiratory diseases. Specifically, we seek to: (i) highlight the key methodological, clinical, and organizational barriers currently limiting the effective adoption of telemedicine in this field; (ii) emphasize the urgent need for the development and adoption of a shared Core Outcome Set (COS) to standardize the evaluation of telemedicine interventions in pediatric respiratory care; and (iii) encourage collaboration among clinicians, researchers, policymakers, technology developers, and patient advocacy groups to support evidence-based, equitable, and sustainable digital care pathways. By addressing these priorities, this review aims to contribute to the evolution of telemedicine from a collection of fragmented pilot initiatives into an integrated and reliable component of standard care for children and adolescents living with chronic respiratory diseases. Given the breadth and heterogeneity of the existing literature, this review was designed as a narrative synthesis rather than a systematic review. Telemedicine interventions in pediatric chronic respiratory diseases encompass a wide range of technologies, care models, and clinical contexts, with substantial variability in study design, outcome selection, follow-up duration, and comparator conditions. Most importantly, outcomes are inconsistently defined and measured across studies, limiting comparability and undermining the validity of quantitative pooling. In this context, a systematic review with or without meta-analysis would be unlikely to generate robust or generalizable effect estimates and could risk overinterpreting fragmented evidence. A narrative approach therefore allows for a more integrative and critical appraisal of the field, enabling the identification of cross-cutting methodological, regulatory, and implementation challenges, and supporting the formulation of a forward-looking framework—centered on outcome harmonization and coordinated implementation—to guide future research and policy development.

## 2. The Imperative for Telemedicine in Pediatric Respiratory Care

Children with chronic respiratory diseases require care models that are continuous, flexible, and multidimensional. For affected patients and their families, the burden of disease extends well beyond respiratory symptoms. Children with chronic respiratory disease often experience recurrent exacerbations, limitations in physical activity, and substantial disruptions to schooling, with cumulative effects on psychosocial development, anxiety, and social participation [1,2,3]. Caregivers, in turn, face significant challenges, including frequent work absences, uncertainty in recognizing early signs of deterioration, and the ongoing emotional and logistical demands of coordinating care across multiple healthcare providers [1,2,3]. Traditional outpatient models—largely episodic and centered on scheduled in-person visits—are poorly suited to address the dynamic and fluctuating nature of these conditions or to provide timely support between clinical encounters.

Telemedicine offers a paradigm shift toward more adaptive, responsive, and patient-centered care [13,14,15,16,17,18]. Digital symptom-monitoring applications can facilitate early identification of changes in respiratory status, enabling proactive clinical interventions before overt decompensation occurs. Technologies designed to support adherence—such as reminder systems and connected inhaler devices—can address one of the most persistent challenges in pediatric respiratory care. Educational platforms and telecoaching interventions have the potential to improve health literacy, promote self-management skills, and empower both patients and caregivers. Moreover, home-based pulmonary function testing and remote physiologic monitoring can support earlier detection of clinical deterioration, reduce unnecessary travel, and optimize resource utilization. For children requiring non-invasive or invasive ventilation, video-assisted home follow-ups and remote troubleshooting may enhance caregiver confidence and reduce avoidable emergency department visits and hospital admissions.

Evidence from diverse international settings suggests that, when appropriately implemented, telemedicine interventions can improve disease control and reduce healthcare utilization. In pediatric asthma, remote monitoring and digital self-management tools have been associated with improved symptom control, reduced reliance on rescue medications, and higher Asthma Control Test (ACT/cACT) scores [19,20,21,22,23,24,25,26,27,28,29,30,31,32,33,34,35,36,37,38,39,40,41,42,43,44,45,46,47,48,49,50,51,52,53,54,55,56,57,58,59,60,61,62,63,64,65,66,67,68,69,70]. In CF, telehealth-enabled exercise and rehabilitation programs have demonstrated potential benefits in lung function, physical performance, and quality of life [71,72,73,74,75,76,77,78,79,80,81,82,83,84,85,86,87,88,89,90,91]. However, despite these encouraging signals, the systematic integration of telemedicine into routine pediatric respiratory care remains limited. This gap reflects not only the relatively modest size of the evidence base but, critically, its methodological fragmentation, characterized by heterogeneous interventions, inconsistent outcome measures, and variable study designs—factors that collectively impede scalability, comparability, and widespread adoption.

## 3. Current Evidence Landscape and Methodological Limitations

Systematic reviews examining telemedicine interventions in pediatric respiratory care consistently reveal a body of evidence characterized by low or very low certainty. Across conditions such as asthma, cystic fibrosis (CF), and chronic respiratory failure, study quality is frequently undermined by methodological weaknesses, including small sample sizes, short follow-up durations, inadequate randomization procedures, and limited or absent blinding. A substantial proportion of published studies are observational in nature or pilot trials lacking appropriate control groups, while many randomized studies are insufficiently powered to detect clinically meaningful differences. As a consequence, meta-analyses are uncommon and, when attempted, are often inconclusive due to pronounced heterogeneity in study populations, intervention modalities, and outcome definitions.

Even in studies reporting favorable effects, the transferability of findings to broader clinical contexts remains uncertain. Many investigations are conducted within single centers or highly specific healthcare systems, thereby limiting external validity and generalizability. Follow-up periods are frequently shorter than six months, precluding meaningful assessment of long-term outcomes such as exacerbation rates, sustained disease control, or avoidance of hospitalizations. Furthermore, economic evaluations are rarely incorporated, leaving critical questions regarding cost-effectiveness and long-term sustainability unanswered [92]. Equity-related considerations—including digital access, health literacy, and socioeconomic determinants of engagement—are similarly underrepresented, despite their central relevance to pediatric populations and family-centered care [93].

Crucially, the absence of standardization in trial design, intervention characteristics, and outcome selection represents a major barrier to evidence synthesis and policy translation. While signals of benefit have been reported—particularly in relation to asthma symptom control and the maintenance of physical activity in CF—these findings cannot be readily compared across studies or translated into robust clinical guidelines, reimbursement frameworks, or large-scale implementation strategies. In this context, outcome heterogeneity should not be viewed as a secondary methodological shortcoming, but rather as a fundamental structural limitation that constrains the generation of high-quality, actionable evidence and impedes the advancement of telemedicine from experimental use to routine clinical practice.

## 4. Why Outcome Heterogeneity Undermines Progress

The evaluation of any medical intervention depends on the use of consistent, validated, and clinically meaningful outcomes [94,95]. Standardized outcomes enable comparison across studies, support the conduct of systematic reviews and meta-analyses, facilitate clinical interpretation, and ultimately inform guideline development, reimbursement decisions, and health policy. In the field of telemedicine for pediatric respiratory care, however, outcome measurement remains highly heterogeneous, both in terms of which outcomes are selected and how they are defined, measured, and reported.

In pediatric asthma studies, for example, disease “control” may be assessed using Asthma Control Test (ACT or cACT) scores, symptom diaries, frequency of rescue medication use, or composite endpoints, often without consistent thresholds, measurement intervals, or definitions of clinical relevance [15,16,17,18,19,20,21,22,23,24,25,26,27,28,29,30,31,32,33,34,35,36,37,38,39,40,41,42,43,44,45,46,47,48,49,50,51,52,53,54,55,56,57,58,59,60,61,62,63,64,65,66,67,68,69,70]. Definitions of exacerbations vary substantially, ranging from hospital admissions to oral corticosteroid prescriptions or caregiver-reported symptom worsening, with limited alignment across studies. In cystic fibrosis and bronchiectasis, lung function outcomes such as FEV_1_, FVC, or peak expiratory flow rate (PEFR) are measured inconsistently, frequently without standardized testing protocols or predefined follow-up schedules [71,72,73,74,75,76,77,78,79,80,81,82,83,84,85,86,87,88,89,90,91]. In other cases, interventions are evaluated using patient- or caregiver-reported outcomes—such as satisfaction or perceived benefit—without the use of validated instruments. Similarly, studies focused on home mechanical ventilation often rely on pragmatic but non-standardized endpoints, including the number of avoided home visits or caregiver confidence, which, while clinically relevant, lack comparability across settings and healthcare systems [96,97,98].

This pervasive inconsistency severely limits the interpretability and usability of the available evidence. Without harmonized outcomes, meaningful aggregation of data is rarely feasible, and meta-analytic synthesis becomes methodologically unsound. As a result, health systems lack the robust, comparable evidence needed to inform clinical pathways, reimbursement models, and investment decisions. Researchers are constrained in their ability to generate cumulative knowledge, and clinicians are left without clear guidance regarding which telemedicine interventions are effective, for which patient populations, and under what conditions. In this context, outcome heterogeneity emerges not as a minor methodological flaw, but as a fundamental impediment to progress, undermining the translation of telemedicine from promising innovation to evidence-based standard of care in pediatric respiratory medicine.

Addressing this structural fragmentation requires a deliberate shift toward standardization. Without a shared framework defining which outcomes should be measured, how they should be assessed, and over what time horizon, future telemedicine studies will continue to generate isolated and non-comparable findings. The development and adoption of a COS for telemedicine interventions in pediatric chronic respiratory diseases therefore represents a critical next step—providing a common evaluative foundation to strengthen evidence synthesis, guide clinical decision-making, and support scalable, policy-relevant implementation.

Although the present review focuses on telemedicine and digital health technologies in pediatric chronic respiratory diseases, the challenges identified are not confined to this clinical area. Evidence from other medical disciplines—including cardiology, diabetes, mental health, oncology, and primary care—consistently demonstrates substantial heterogeneity in telemedicine interventions, outcome definitions, follow-up duration, and comparators. This variability has frequently resulted in inconsistent findings across studies and has markedly limited the feasibility and interpretability of meta-analyses. While meta-analytic data are available in selected fields, these analyses often report low to very low certainty of evidence, high statistical heterogeneity, and context-dependent effects, underscoring the difficulty of drawing generalizable conclusions about clinical effectiveness. Consequently, the broader telemedicine literature suggests that the lack of standardized outcome frameworks represents a cross-disciplinary methodological barrier rather than a disease-specific limitation. These observations reinforce the need for harmonized core outcome sets not only in pediatric respiratory care but across telemedicine research more broadly, to enable meaningful evidence synthesis, support health technology assessment, and facilitate the integration of digital health interventions into routine clinical practice.

## 5. Proposal: A Core Outcome Set for Telemedicine in Pediatric Respiratory Care

Although asthma, cystic fibrosis, bronchiectasis, and chronic respiratory failure differ substantially in disease trajectory, monitoring intensity, and clinical management, they share a set of common care challenges that are particularly amenable to telemedicine-based solutions. Across these conditions, care is longitudinal, requires close monitoring between in-person visits, depends heavily on treatment adherence and caregiver engagement, and is sensitive to early detection of clinical deterioration. The proposed telemedicine framework and COS are therefore not intended to replace disease-specific outcomes or management strategies, but to define a minimum, cross-cutting evaluative foundation that captures outcomes relevant across chronic pediatric respiratory conditions. Core domains such as exacerbation-related healthcare utilization, lung function trends, treatment adherence, health-related quality of life, school absenteeism, and caregiver burden reflect shared dimensions of disease impact and system-level value, while allowing condition-specific extensions (e.g., CF-specific biomarkers, ventilation parameters, or bronchiectasis imaging scores) to be incorporated as supplementary outcomes. This layered approach—combining a shared core with disease-tailored measures—supports comparability and evidence synthesis without oversimplifying clinical heterogeneity, and aligns with established COS methodology in other heterogeneous disease areas.

To overcome the foundational barrier of the COS absence in pediatric pulmonology, we call for the urgent development and widespread adoption of a COS for telemedicine interventions in pediatric chronic respiratory diseases. A COS represents an agreed, standardized minimum set of outcomes that should be measured and reported across all clinical studies within a defined field. Its systematic use ensures that research generates comparable, clinically meaningful, and policy-relevant evidence, thereby facilitating evidence synthesis, informing guideline development, and supporting regulatory and reimbursement decisions.

We propose that the COS for pediatric respiratory telemedicine should include a limited number of outcomes selected on the basis of clinical relevance, feasibility across healthcare settings, sensitivity to change, and alignment with patient- and family-centered priorities (Table 1).

The following domains are proposed as core outcomes:

1. Exacerbation Frequency, defined as acute respiratory events requiring emergency department presentation or hospitalization, recorded prospectively and verified through clinical documentation.
2. Lung Function, assessed using standardized measures such as forced expiratory volume in one second (FEV_1_) expressed as percentage of predicted values and peak expiratory flow rate (PEFR), obtained with validated, age-appropriate devices in clinical or home-based settings.
3. Healthcare Utilization, including the number of respiratory-related emergency visits, hospital admissions, and intensive care unit admissions, captured over a minimum follow-up period of 12 months.
4. Treatment Adherence, quantified through objective measures such as electronic monitoring devices (e.g., smart inhalers) or pharmacy refill data, rather than self-report alone.
5. Health-Related Quality of Life (HRQoL), measured using validated generic and disease-specific instruments, such as the Pediatric Quality of Life Inventory (PedsQL), the Cystic Fibrosis Questionnaire–Revised (CFQ-R), or the Pediatric Asthma Quality of Life Questionnaire (PAQLQ).
6. School Absenteeism, expressed as the number of school days missed per academic term due to respiratory illness, as reported by caregivers or educational institutions.
7. Caregiver Burden, assessed using validated tools such as the Caregiver Strain Index or the Family Impact Module, capturing the broader psychosocial and practical impact of chronic respiratory disease management.

For each outcome, standardized definitions, measurement instruments, data collection procedures, and recommended assessment intervals should be explicitly specified. The final COS should be established through a transparent, consensus-based process—such as a Delphi methodology—actively involving clinicians, researchers, patients, caregivers, policymakers, and digital health experts. To ensure meaningful uptake, we advocate that funding agencies, ethics committees, and scientific journals require the use of this COS in future telemedicine trials, thereby raising methodological quality, enhancing comparability, and accelerating the translation of digital innovations into routine pediatric respiratory care.

**Figure 1:**
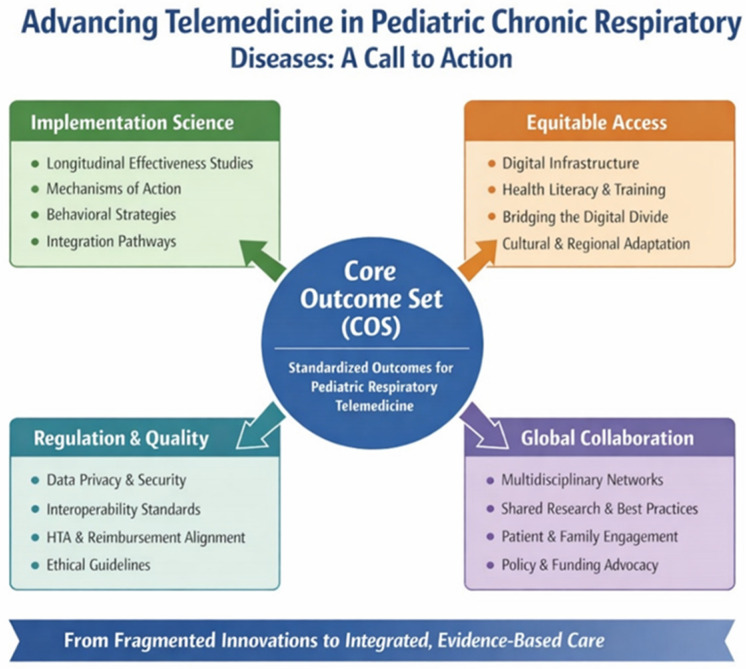
Conceptual framework for advancing telemedicine in pediatric chronic respiratory diseases.

Figure 1 shows the key pillars required to transform telemedicine from fragmented pilot initiatives into an integrated, evidence-based model of care for pediatric chronic respiratory diseases. A COS is positioned at the center as the foundational element enabling standardized evaluation, comparability across studies, and translation into clinical practice and policy. Four interconnected domains surround the COS: (1) Implementation Science, supporting real-world adoption, sustainability, and scalability; (2) Equitable Access, addressing the digital divide through infrastructure, literacy, and inclusive design; (3) Regulation and Quality Assurance, ensuring safety, interoperability, ethical governance, and alignment with Health Technology Assessment and reimbursement processes; and (4) Global Collaboration, promoting shared standards, data harmonization, and patient- and family-centered innovation. Together, these elements support the transition from isolated digital tools to coordinated, sustainable, and equitable telemedicine pathways in pediatric respiratory care.

The COS proposed in this review should be interpreted as a preliminary, concept-generating framework rather than a finalized or prescriptive standard. Its purpose is to make explicit the need for outcome harmonization in pediatric respiratory telemedicine and to provide a clinically informed starting point for structured consensus-building. We acknowledge that rigorous COS development requires systematic stakeholder engagement, including children, adolescents, caregivers, clinicians, researchers, health economists, policymakers, and digital health experts, as well as alignment with established COS development initiatives. Accordingly, the COS outlined here is intended to be refined through a transparent, multi-step process encompassing stakeholder mapping, patient and caregiver involvement, feasibility assessment across diverse healthcare settings, and formal consensus methodologies such as Delphi surveys and consensus meetings. Outcome prioritization should be informed not only by clinical relevance but also by patient- and family-centered value, measurement feasibility, sensitivity to change, and relevance to health technology assessment and reimbursement decisions. By explicitly positioning the COS as an evolving, consensus-driven construct, we aim to avoid prescriptiveness and instead invite coordinated international collaboration to develop a robust, widely accepted outcome framework capable of supporting meaningful evidence synthesis and sustainable telemedicine implementation in pediatric chronic respiratory diseases.

The harmonization strategy proposed in this review is not intended to operate in isolation, but rather to align with and build upon existing international initiatives in digital health evaluation, pediatric outcomes research, and telemedicine governance. Established frameworks from regulatory authorities and methodological consortia—such as international efforts in COS development, guidance on digital health evaluation and health technology assessment, and standards for software as a medical device—provide essential foundations for safety, effectiveness, and interoperability. However, these initiatives have largely evolved in parallel, with limited integration at the level of disease-specific, pediatric, and telemedicine-focused outcome evaluation. The proposed COS is therefore positioned as a translational interface between these frameworks, operationalizing their principles within the specific context of pediatric chronic respiratory care. By explicitly linking clinical outcomes, patient- and caregiver-reported measures, implementation metrics, and system-level indicators, this approach seeks to complement—not duplicate—existing regulatory and methodological standards, while facilitating comparability across studies, jurisdictions, and care models. Embedding telemedicine evaluation within this aligned, multi-layered ecosystem is essential to avoid fragmentation and to ensure that digital innovations progress coherently from pilot studies to regulated, reimbursable, and sustainable standards of care.

## 6. Addressing the Digital Divide and Health Equity

While telemedicine can expand access to pediatric respiratory care, its implementation may reinforce existing health inequities unless equity is deliberately embedded in policy, design, and evaluation frameworks. Evidence from Europe and North America shows that families with lower socioeconomic status, immigrant and minority backgrounds, and those living in rural or underserved areas often lack reliable broadband access, appropriate devices, or sufficient digital literacy to engage effectively with telehealth services [93]. These barriers are particularly relevant in pediatric respiratory care, where disease burden frequently overlaps with structural disadvantage, including in socioeconomically deprived regions of Italy and other healthcare systems.

Addressing these disparities requires coordinated national and regional action. Telemedicine should be integrated into publicly funded healthcare systems with reimbursement models that incentivize equitable delivery rather than selective adoption. Coverage should include teleconsultations, remote monitoring, and digital education, while accounting for the additional time needed to support families with limited digital literacy. Digital inclusion strategies—such as universal broadband access, subsidized devices, and community-based digital literacy initiatives—should be aligned with healthcare priorities.

Equity must also be embedded in evaluation frameworks. The adoption of a COS offers an opportunity to mandate systematic collection of sociodemographic variables, enabling stratified analyses of effectiveness and engagement. Outcomes such as healthcare utilization, school absenteeism, treatment adherence, and caregiver burden should be examined across population subgroups to detect differential effects and unintended disparities.

Finally, user-centered and co-design approaches are essential to ensure that digital tools are usable, culturally appropriate, and accessible across languages, literacy levels, and developmental stages. Adolescents require particular attention given their specific needs related to autonomy, privacy, and digital engagement. Recognizing equity as a core quality domain—and aligning reimbursement, digital inclusion, and standardized outcome measurement through the COS—will be critical to ensuring that telemedicine reduces rather than widens health disparities for children with chronic respiratory diseases.

## 7. Economic Sustainability and the Case for Cost-Effectiveness

Telemedicine is often promoted as a cost-saving innovation; however, robust economic evidence remains limited, particularly in pediatric respiratory care [99]. From the perspective of healthcare payers and Health Technology Assessment (HTA) agencies, claims of economic value require transparent, reproducible, and context-sensitive evaluation. Although potential cost offsets may result from fewer unplanned hospitalizations, emergency department visits, travel requirements, and caregiver work absences, these savings must be weighed against the direct and indirect costs of implementation, including platform development and maintenance, device procurement, data storage and cybersecurity, integration with electronic health records, and clinician time for data review and follow-up.

To inform reimbursement and coverage decisions, future telemedicine studies should incorporate prospective economic evaluations aligned with HTA standards. Analyses should adopt a societal perspective, capturing not only healthcare expenditures but also broader economic consequences for children and families, such as caregiver productivity, transportation costs, school absenteeism, and potential long-term benefits from improved disease control. Where feasible, evaluations should extend beyond short-term horizons to reflect the chronic nature of pediatric respiratory diseases.

The adoption of a COS provides a critical opportunity to strengthen the economic evidence base. Standardized measurement of outcomes such as exacerbation frequency, healthcare utilization, treatment adherence, school attendance, and caregiver burden would enable more comparable cost-effectiveness analyses across studies and jurisdictions—an essential requirement for HTA, pricing, and value-based reimbursement decisions.

Without high-quality economic evidence aligned with HTA requirements, telemedicine is likely to remain confined to pilot initiatives or temporary funding mechanisms. Embedding rigorous, standardized economic evaluation into telemedicine research is therefore essential to support sustainable reimbursement models and to position digital care as a credible, value-based component of pediatric respiratory healthcare systems.

## 8. Regulation, Accreditation, and Quality Assurance

The rapid expansion of telemedicine has created a crowded and uneven digital health landscape, characterized by wide variability in clinical validation, regulatory oversight, and quality assurance. Many telemedicine platforms have been developed outside traditional medical device pathways and introduced into practice without formal certification, resulting in limited compliance with requirements for clinical safety, interoperability, and data protection [100,101,102]. These gaps are particularly concerning in pediatric care, where age-specific privacy protections and usability standards are essential.

Regulatory authorities should therefore clarify and enforce the application of existing medical device frameworks to telemedicine solutions. In the European Union, platforms with diagnostic or therapeutic functions should be assessed under the Medical Device Regulation (MDR) and, where applicable, obtain CE marking to demonstrate conformity with safety, performance, and post-market surveillance requirements [101]. Similarly, in the United States, telemedicine software qualifying as Software as a Medical Device (SaMD) falls under the regulatory oversight of the Food and Drug Administration (FDA), including its digital health and precertification programs [102]. Clear guidance on classification and risk stratification is needed to reduce uncertainty for developers and to protect patients and clinicians.

Beyond regulatory approval, health systems should adopt structured accreditation processes for telemedicine platforms intended for routine use. These should assess not only regulatory compliance but also clinical effectiveness, cybersecurity, data protection (including GDPR adherence), and interoperability with electronic health records to ensure continuity of care and integration into clinical workflows.

Quality assurance must also address usability, accessibility, and equity. Accreditation should explicitly evaluate user experience for clinicians and families, including age-appropriate design, accessibility for users with disabilities or limited digital literacy, multilingual support, and pediatric-specific consent processes. Certification should be accompanied by ongoing post-market surveillance and real-world performance monitoring to ensure sustained safety and effectiveness as platforms scale.

Finally, regulatory approval and accreditation should be aligned with reimbursement and HTA processes [103]. Platforms lacking CE marking, FDA clearance, or equivalent validation should not be eligible for routine reimbursement, whereas certified solutions demonstrating clinical benefit, interoperability, and equitable usability should be prioritized for coverage. Aligning regulation, accreditation, and reimbursement is essential to move telemedicine from a fragmented digital marketplace to a trusted and sustainable component of pediatric respiratory care.

## 9. Implementation Science and Real-World Adoption

Translating evidence into routine clinical practice requires more than demonstration of efficacy under controlled conditions (Table 2).

It necessitates a systematic understanding of the real-world contexts in which telemedicine interventions are introduced, including organizational constraints, workforce capacity, technological readiness, and patient engagement [13,16]. Implementation science offers established conceptual frameworks and methodological approaches to examine how, why, and under what conditions telemedicine interventions are adopted, implemented, and sustained in routine pediatric respiratory care [16]. Key implementation outcomes—such as fidelity, feasibility, acceptability, penetration, and sustainability—should therefore be considered essential complements to traditional clinical endpoints [16].

Hybrid effectiveness–implementation study designs should be prioritized to concurrently evaluate clinical outcomes and implementation processes [16]. Such approaches enable early identification of barriers and facilitators to adoption, reducing the risk that clinically effective interventions fail during scale-up. Implementation outcomes should be measured using standardized tools where available and reported transparently to facilitate cross-study learning and replication across healthcare systems [12,16].

Mixed-methods research is central to this effort. Quantitative data on clinical effectiveness, healthcare utilization, and treatment adherence should be integrated with qualitative insights from patients, caregivers, and healthcare professionals [12]. These data can elucidate critical factors such as workflow integration, workforce burden, role adaptation, technical reliability, digital literacy challenges, and perceived value of telemedicine tools, which are essential for acceptability and long-term sustainability [13,16].

Finally, implementation research should be explicitly linked to health equity and system-level outcomes. Evaluations should examine differential adoption and effectiveness across sociodemographic groups, care settings, and geographic regions to ensure that telemedicine does not exacerbate existing disparities [93]. Embedding implementation science alongside standardized outcome measurement frameworks can therefore generate actionable evidence to support scalable, equitable, and durable integration of digital care into pediatric respiratory practice [13,16].

## 10. Toward Global Coordination: A Collaborative Future

We acknowledge that many of the recommendations advanced in this review—particularly those concerning reimbursement, regulation, accreditation, and health technology assessment—are not yet underpinned by a robust, pediatric-specific evidence base. The current literature is characterized by heterogeneous interventions, variable outcome definitions, and predominantly low to very low certainty of evidence, which limits the ability to draw definitive conclusions regarding cost-effectiveness or long-term system impact. Accordingly, the policy-oriented recommendations presented here should be interpreted as conditional and principle-based rather than as endorsements for immediate, large-scale implementation. Their intent is to outline the governance and evaluation prerequisites required for responsible scale-up, should telemedicine interventions demonstrate sufficient clinical value when assessed using standardized outcomes and rigorous economic frameworks. By explicitly linking future reimbursement and regulatory decisions to the generation of harmonized, pediatric-relevant evidence—through standardized outcome measurement, prospective economic evaluation, and real-world implementation studies—we aim to align telemedicine policy development with the evidentiary standards expected by policymakers and HTA agencies, while avoiding premature or unsupported system-wide adoption.

Sustained progress in pediatric respiratory telemedicine will remain limited as long as research, policy development, and implementation efforts continue to occur in isolation within individual countries, institutions, or healthcare systems [12,16]. Fragmented initiatives risk duplicating efforts, perpetuating methodological inconsistencies, and slowing the generation of robust, generalizable evidence [15,16]. To overcome these limitations, we call for the establishment of a coordinated international collaborative dedicated to telemedicine in pediatric chronic respiratory diseases.

Such a collaborative should bring together clinicians, researchers, health system leaders, policymakers, HTA agencies, digital health developers, and patient advocacy organizations [4,12,13]. Its core functions should include the coordinated development, refinement, and global dissemination of a shared COS; the promotion of interoperable data standards; and the facilitation of secure and ethical sharing of de-identified patient-level data across jurisdictions [4,16]. In addition, the consortium should endorse evidence-based implementation science frameworks, support comparative effectiveness research, and enable cross-country learning on regulatory, reimbursement, and organizational models that facilitate sustainable telemedicine adoption [13,16].

Equally importantly, this collaborative should institutionalize the meaningful involvement of children, adolescents, and their families. Embedding patient and caregiver perspectives within governance structures, research prioritization, and outcome selection is essential to ensure that digital innovations reflect real-world needs, preferences, and lived experiences [14,20]. By providing a formal platform for co-design and shared accountability, a global consortium can accelerate the translation of telemedicine from isolated pilot initiatives into harmonized, equitable, and evidence-based care pathways [12,13].

Ultimately, global coordination offers an opportunity not only to improve methodological rigor and efficiency but also to align innovation with shared values of equity, transparency, and patient-centeredness [15,16]. Through collective action, pediatric respiratory telemedicine can evolve into a mature, collaborative field capable of delivering scalable and meaningful improvements in care for children with chronic respiratory diseases worldwide.

## 11. Historical Inertia and the Need for Paradigm Shift

Telemedicine is not a novel concept in pediatric respiratory care. For decades, clinicians have explored remote approaches ranging from home pulse oximetry and telephone-based asthma education to early forms of remote symptom reporting [11,20]. Nevertheless, these strategies have largely remained peripheral to standard care, implemented mainly as adjuncts, pilot initiatives, or short-term projects rather than as integrated, system-level solutions [12,16]. This historical inertia reflects a combination of structural, cultural, and economic barriers that have limited the translation of innovation into routine practice [15,16].

Traditional reimbursement models have favored in-person encounters, providing little incentive to invest in remote care [99]. In parallel, inadequate digital infrastructure and limited interoperability have discouraged institutional adoption [13,16]. Professional skepticism has also contributed, as clinicians trained within face-to-face care paradigms have been cautious about relying on digital proxies for clinical assessment, particularly in pediatric populations [14,17]. Healthcare organizations have likewise been reluctant to commit resources to platforms lacking regulatory validation, reimbursement pathways, or a robust evidence base [100,101,102]. Even as studies suggested potential clinical benefits, the absence of standardized outcome frameworks undermined comparative effectiveness assessments and limited policymakers’ ability to justify sustained investment [15,16].

The COVID-19 pandemic temporarily altered this landscape, accelerating telemedicine adoption as regulatory constraints were relaxed and remote care became essential to maintain continuity of services [7,17]. During this period, telemedicine demonstrated feasibility at scale and the ability to support children and families during unprecedented system strain [14,16]. However, in many settings, this momentum proved fragile; as emergency measures were withdrawn and reimbursement policies reverted, telemedicine use declined, with a return to predominantly in-person care models [7,16].

This reversion should not be interpreted as evidence of telemedicine’s ineffectiveness. Rather, it reflects a failure to consolidate pandemic-era experience into a durable, evidence-based transformation of care delivery [15,16]. Without sustained policy support, standardized outcome measurement, and rigorous, reproducible evaluation frameworks, telemedicine remains vulnerable to cyclical, crisis-driven adoption [12,16]. A genuine paradigm shift will require reframing telemedicine not as a temporary substitute for in-person care, but as an integral, evidence-based component of pediatric respiratory care—designed, evaluated, and reimbursed with the same rigor as any other medical intervention [4,13,16].

## 12. Learning from Success: Exemplars of Pediatric Telehealth

Despite the structural and methodological challenges that continue to limit widespread adoption, a growing number of real-world success stories demonstrate what pediatric respiratory telehealth can achieve when thoughtfully designed and systemically supported [7,9,10,12]. Pediatric centers across Europe, North America, and Australia have developed integrated telemedicine pathways for asthma and cystic fibrosis management, reporting clinically meaningful outcomes and operational sustainability [8,9,10,18]. Table 3 shows telemedicine tools according to pediatric respiratory conditions suitable for online monitoring.

What distinguishes successful initiatives is not the technology itself, but its integration within coherent models of care. Effective programs combine digital tools with sustained human support, clearly defined clinical responsibilities, and continuous feedback loops that allow timely adjustment of care plans [4,13,16]. Importantly, these initiatives embed telemedicine within existing care pathways rather than positioning it as a parallel or optional add-on, and they rely on systematic collection of clinically meaningful outcomes to support ongoing evaluation, iterative refinement, and demonstration of value to clinicians, administrators, and payers [15,16].

Collectively, these experiences underscore that effective pediatric telehealth depends on co-design with patients and families, interoperability with health system infrastructure, sensitivity to local context, and standardized outcome measurement [4,12,13]. When implemented as part of an integrated, evidence-driven strategy rather than as an isolated technological solution, telemedicine can deliver durable improvements in care quality, efficiency, and equity [15,16]. These lessons provide a practical blueprint for scaling telemedicine beyond isolated centers toward harmonized, system-wide adoption.

## 13. The Danger of a Fragmented Digital Ecosystem

In the absence of strategic coordination, telemedicine risks evolving into a fragmented digital ecosystem composed of disconnected applications, devices, and platforms [12,16]. Commercial innovation has advanced faster than clinical validation and system-level governance, leaving pediatric pulmonologists faced with a rapidly expanding array of digital tools that vary widely in quality, regulatory status, and evidentiary support [100,101,102]. Many platforms lack formal regulatory approval, robust data protection safeguards, or transparent reporting of clinical outcomes, raising concerns about safety, effectiveness, and accountability—particularly in pediatric care [101,102]. For families, this fragmentation often results in the need to manage multiple, non-interoperable applications with different interfaces, notification systems, and data-sharing requirements, increasing the burden of care [14].

The consequences for clinicians and healthcare organizations are equally substantial. Limited interoperability obliges clinicians to navigate between multiple platforms, increasing cognitive workload, disrupting clinical workflows, and reducing efficiency [13,16]. Data silos hinder longitudinal monitoring, compromise comprehensive clinical assessment, and weaken continuity of care [12,16]. At the health system level, fragmentation obstructs the development of integrated digital care pathways and complicates evaluation of effectiveness, resource allocation, and accountability [15,16].

Beyond operational inefficiencies, fragmentation erodes trust among all stakeholders. Families may be uncertain about which digital tools are clinically endorsed or evidence-based, while clinicians may hesitate to recommend platforms that lack regulatory validation or standardized outcome reporting [14,15]. Developers, in turn, lack clear benchmarks for improvement in the absence of shared outcome frameworks, limiting meaningful comparison and cumulative learning across solutions [16].

The solution is not to restrict innovation, but to guide it through coherent standards and governance mechanisms. Regulatory authorities and health systems should require not only compliance with safety, privacy, and interoperability standards, but also demonstration of clinical impact using harmonized COS [101,102,103]. Aligning regulatory approval, accreditation, reimbursement eligibility, and outcome measurement is essential to transform telemedicine from a collection of isolated digital products into a coordinated, trustworthy, and learning digital ecosystem capable of supporting high-quality pediatric respiratory care at scale [15,16].

Without such alignment, fragmentation will continue to undermine trust in telehealth, confuse families, and impede the development of coherent digital care models. Conversely, embedding standardized outcome frameworks within regulatory and reimbursement processes can provide developers with clear performance benchmarks and reassure patients and clinicians that digital interventions are evidence-based and clinically meaningful [15,16].

While political commitment is clearly necessary to enable the sustainable integration of telemedicine into pediatric respiratory care, such commitment must be translated into concrete and operational actions to be effective. Beyond high-level policy endorsement, this includes the allocation of dedicated funding for digital infrastructure and interoperability, the establishment of clear reimbursement pathways linked to defined clinical and outcome criteria, and the development of accreditation and training standards for healthcare professionals delivering telemedicine services. Practical implementation also requires investment in workforce capacity, including protected clinician time for remote care activities, as well as governance frameworks that define responsibility for data review, escalation pathways, and medico-legal accountability. At the system level, the incorporation of standardized outcomes into routine data collection and health technology assessment processes is essential to inform adaptive reimbursement and regulatory decisions. Framing political commitment in these pragmatic terms helps bridge the gap between policy intent and clinical reality, ensuring that telemedicine initiatives are not only endorsed but also implementable, equitable, and sustainable in everyday pediatric respiratory practice.

## 14. Ethical Considerations in Pediatric Telemedicine

Telemedicine in pediatric care raises ethical considerations that are distinct from those encountered in adult populations and require dedicated attention [13,14]. Children and adolescents differ in their cognitive, emotional, and developmental capacities, which influence their ability to understand, engage with, and consent to digital health interventions. While adolescents may value autonomy, privacy, and digital communication, younger children typically depend on caregiver mediation for decision-making and technology use. Ethical implementation must therefore account for evolving capacities, balancing the child’s right to participation with appropriate parental involvement [14,17].

Data privacy and confidentiality represent particularly sensitive ethical domains in pediatric telemedicine. Digital platforms often collect large volumes of personal and health-related data, including passively generated data from applications, sensors, or wearable devices, raising concerns about proportionality, data minimization, secondary use, and long-term storage [100,101,102]. These issues are especially relevant when data are generated continuously outside traditional clinical encounters. Excessive monitoring may also lead to over-surveillance, potentially increasing anxiety, altering behavior, or undermining a child’s sense of autonomy and trust. Ethical telemedicine design should therefore prioritize transparency, purpose limitation, and protection of children’s digital well-being [101,102].

Equity considerations further reinforce the ethical dimension of telemedicine deployment. When access to digital health tools depends on financial resources, technological skills, or stable internet connectivity, existing social and health inequalities may be exacerbated [93]. Children from socioeconomically disadvantaged or marginalized communities may face a dual burden of higher chronic respiratory disease prevalence and reduced access to digitally mediated care, challenging core ethical principles of justice and fairness in pediatric healthcare [16,93].

Accordingly, the expansion of telemedicine in pediatric respiratory care must be guided by explicit ethical frameworks tailored to children and adolescents. These should address age-appropriate consent and assent processes, robust data protection and governance, proportional use of monitoring technologies, promotion of digital well-being, and proactive measures to prevent technology-driven inequities [13,14,16]. Embedding ethical oversight within regulatory, accreditation, and evaluation processes is essential to ensure that telemedicine advances not only clinical innovation, but also the rights, dignity, and best interests of children and their families [101,102,103].

While telemedicine offers important opportunities to enhance continuity of care and patient engagement, its implementation is not without potential risks and unintended consequences that warrant careful consideration. Excessive or poorly targeted remote monitoring may lead to over-surveillance, data fatigue for families, and increased anxiety or medicalization of everyday life, particularly in pediatric settings where caregivers already shoulder substantial responsibility. From a clinical perspective, continuous data streams can escalate clinician workload, contribute to alert fatigue, and divert attention from clinically meaningful signals if not supported by clear thresholds, triage protocols, and adequate staffing. There is also a risk of false reassurance when remotely collected data are incomplete, inaccurately measured, or interpreted outside appropriate clinical context, potentially delaying necessary in-person assessment. These challenges highlight that telemedicine should not be viewed as a simple technological add-on, but as a complex care intervention requiring thoughtful design, proportional monitoring strategies, integration into clinical workflows, and explicit safeguards to preserve clinical judgment and family well-being. Acknowledging and addressing these limitations is essential to ensure that telemedicine enhances—rather than inadvertently compromises—the quality, safety, and humanity of pediatric respiratory care.

## 15. Expanding the Research Agenda: Beyond the Core Outcome Set

Telemedicine offers several potential advantages for the management of pediatric chronic respiratory diseases. These include improved access to specialist care, particularly for families in remote or underserved areas; enhanced continuity of care between scheduled visits; earlier recognition of symptom deterioration or exacerbations; support for treatment adherence and self-management; and reductions in travel burden, school absenteeism, and caregiver time costs. Telemedicine may also facilitate patient and caregiver education and promote more proactive, patient-centered care models. However, these benefits must be weighed against important limitations and risks. Potential disadvantages include over-monitoring and data overload, increased clinician workload and alert fatigue, variable data quality from home-based measurements, and the risk of false reassurance or delayed escalation to in-person care. For families, intensive digital monitoring may contribute to anxiety, digital fatigue, or medicalization of home life, while disparities in digital access and literacy may exacerbate existing health inequities. Recognizing both the strengths and limitations of telemedicine is essential to guide proportionate, context-sensitive implementation and to ensure that digital tools complement—rather than replace—clinical judgment and in-person care. Table 4 summarizes advantages and disadvantages of online consultations in pediatric chronic respiratory care.

While the development and adoption of a COS is a critical prerequisite for improving methodological rigor and comparability, it represents only the foundation of a broader research agenda required to advance telemedicine in pediatric respiratory care. To support durable, evidence-based integration into clinical practice, future research must extend beyond short-term efficacy and address the complex, longitudinal, behavioral, and organizational dimensions of digital care (Table 5).

First, longitudinal effectiveness studies are urgently needed to determine how telemedicine interventions influence disease trajectories over extended time horizons. Evaluations spanning two to five years are essential to assess sustained effects on exacerbation frequency, lung function decline, healthcare utilization, and transition outcomes from pediatric to adult care. Such studies would clarify whether early digital interventions modify long-term disease burden or merely produce transient improvements.

Second, research should focus on elucidating the mechanisms of action underlying observed benefits. Telemedicine interventions are typically multi-component, combining symptom monitoring, automated feedback, education, behavioral prompts, and clinician interaction. Disentangling which components—or combinations thereof—drive clinical and patient-reported outcomes is necessary to optimize intervention design, avoid unnecessary complexity, and improve cost-effectiveness.

Third, insights from behavioral economics and behavioral science should be systematically integrated into intervention development and evaluation. Digital nudges, gamification strategies, incentives, and adaptive feedback may enhance engagement and adherence, particularly among children and adolescents. Rigorous testing is required to determine which behavioral strategies are effective, for whom, and under what conditions, while also assessing potential unintended consequences such as disengagement or digital fatigue.

Fourth, advances in integration and organizational science are essential to understand how telemedicine can be sustainably embedded within healthcare systems. Research should examine the organizational structures, workforce models, reimbursement mechanisms, and digital infrastructure that enable long-term adoption. Comparative studies across health systems can identify scalable models of care delivery and clarify the institutional changes required to support telemedicine beyond pilot phases.

Fifth, equity-focused and disparities research must be embedded as a core component of the research agenda. Studies should routinely assess differential effectiveness, uptake, and engagement across socioeconomic strata, geographic regions, cultural backgrounds, and comorbidity profiles. Such analyses are essential to ensure that telemedicine contributes to reducing—rather than exacerbating—health inequities in pediatric respiratory care.

To advance this agenda, funding agencies and research sponsors should prioritize not only randomized controlled trials but also pragmatic trials, hybrid effectiveness–implementation studies, and health services research. Investment in interdisciplinary research—bridging clinical medicine, behavioral science, health economics, implementation science, and digital health—is essential to generate the evidence required for system-wide adoption. By expanding the research focus beyond outcome standardization alone, the field can move toward telemedicine models that are clinically effective, economically sustainable, organizationally viable, and equitable over the long term.

Beyond asthma and cystic fibrosis, several other pediatric respiratory conditions appear particularly well suited to telemedicine-supported care, provided that interventions are appropriately tailored to disease characteristics and care intensity. Children with non-cystic fibrosis bronchiectasis may benefit from remote symptom tracking, treatment adherence monitoring, and tele-rehabilitation programs to support airway clearance and early detection of infective exacerbations. Patients with chronic respiratory failure, including those requiring long-term oxygen therapy or non-invasive ventilation, are candidates for telemonitoring of device parameters, oxygen saturation, and ventilator performance, combined with structured teleconsultations to optimize settings and reduce unplanned hospital visits. Neuromuscular disorders with respiratory involvement represent another high-impact area, where telemedicine can support longitudinal monitoring of respiratory symptoms, caregiver-reported outcomes, and home ventilation interfaces, alongside remote caregiver training. Children with sleep-disordered breathing, particularly those on long-term positive airway pressure therapy, may benefit from telemonitoring of device adherence and efficacy data, coupled with virtual follow-up visits. Across these conditions, the most useful telemedicine tools include structured symptom-reporting platforms, adherence-monitoring devices, home pulse oximetry and spirometry (where feasible), secure data dashboards with clinically meaningful alerts, and teleconsultations integrated into multidisciplinary care pathways. Identifying disease-specific use cases and matching them with proportionate digital tools is essential to maximize clinical value while minimizing unnecessary monitoring burden.

## 16. Conclusions

Telemedicine holds substantial promise to transform the care of children and adolescents with chronic respiratory diseases by enabling more continuous, personalized, and accessible models of care. However, its future impact will depend not on technological advancement alone, but on the rigor with which telemedicine is evaluated, governed, and integrated into healthcare systems. At present, the lack of standardized outcome measurement represents a critical structural weakness, limiting evidence synthesis, undermining policy translation, and constraining sustainable implementation.

To further enhance the translational value of telemedicine research in pediatric respiratory care, it is essential to explicitly exemplify the standards used, maintain a pragmatic focus on clinically actionable elements, and clearly summarize advantages and disadvantages. From a standards perspective, alignment with existing regulatory, ethical, and methodological frameworks—including data protection requirements, certification of digital tools, and the use of validated clinical and patient-reported outcome measures—provides a concrete foundation for real-world implementation. A pragmatic reading of the literature shows that telemedicine offers clear advantages, such as improved continuity of care, earlier identification of clinical deterioration, reduced travel burden, and enhanced communication with children and families, particularly when combined with structured educational and psychological support interventions. At the same time, important limitations must be acknowledged, including variability in digital access and literacy, the risk of increased clinician workload, potential over-monitoring, and the possibility of reduced effectiveness when telemedicine is not well integrated into existing care pathways. Presenting these strengths and weaknesses in a balanced and concise manner supports informed decision-making by clinicians, health systems, and policymakers, and facilitates the responsible implementation of telemedicine as a complementary—not substitutive—tool in pediatric practice, with particular attention to family engagement and psychosocial well-being.

The available evidence on telemedicine in pediatric chronic respiratory diseases is heterogeneous not only in intervention characteristics and outcome selection, but also in methodological quality and stage of evaluation. The literature is dominated by pilot and feasibility studies, small observational cohorts, and short-term interventions, with relatively few adequately powered randomized controlled trials. Where randomized studies are available, they vary substantially in design, comparators, and outcome definitions, limiting cross-study comparability. Observational studies frequently introduce risks of selection bias, confounding, and performance bias, while pilot studies are primarily designed to assess feasibility and acceptability rather than clinical effectiveness. Although a formal risk-of-bias assessment was not performed, study findings are interpreted throughout the manuscript in light of these methodological differences, and greater weight is placed on evidence from randomized and more mature evaluations when available. This uneven evidence landscape underscores the exploratory nature of much of the current literature and reinforces the need for standardized outcome frameworks and rigorously designed trials to support reliable evidence synthesis and informed implementation.

Addressing these gaps requires a coordinated and sustained commitment to the development and adoption of a COS tailored to telemedicine interventions in pediatric respiratory care. Standardized outcomes are essential to generate comparable, high-quality evidence that can inform clinical guidelines, HTA, regulatory approval, and reimbursement decisions. Yet, outcome harmonization, while necessary, is not sufficient. Telemedicine must be studied and implemented within robust implementation science frameworks, supported by rigorous economic evaluation, ethical oversight, and regulatory alignment to ensure safety, effectiveness, and long-term sustainability.

Equity must be embedded as a foundational principle of digital health transformation. Without deliberate investment in digital infrastructure, literacy, and inclusive design, telemedicine risks widening existing health disparities. Policies and evaluation frameworks must therefore prioritize equitable access and systematically assess differential impact across populations, ensuring that digital innovation benefits those with the greatest burden of disease.

Finally, the advancement of pediatric respiratory telemedicine demands global collaboration. Building international networks that unite clinicians, researchers, policymakers, payers, technology developers, and patient advocacy groups will be essential to harmonize outcomes, share knowledge, and accelerate learning across health systems. Centering the voices of children, adolescents, and families within these collaborations is critical to ensuring that innovation remains grounded in real-world needs and lived experience.

If these priorities are addressed with urgency and vision, telemedicine can move beyond fragmented pilot projects and episodic crisis-driven adoption to become a trusted, evidence-based, and integral component of pediatric respiratory care. By doing so, health systems can deliver care that is not only technologically advanced but also clinically effective, ethically sound, economically sustainable, and responsive to the needs of every child living with chronic respiratory disease.

## Figures and Tables

**Table 1:** Core Outcome Set (COS) Proposed for Telemedicine Interventions in Pediatric Chronic Respiratory Diseases.

Outcome Domain	Definition	Measurement Approach	Rationale
Exacerbation Frequency	Acute respiratory events requiring ED visit or hospitalization	Prospective recording; clinical documentation	Key marker of disease control
Lung Function	FEV_1_ (% predicted) and/or PEFR	Validated clinical or home devices	Objective assessment of disease severity
Healthcare Utilization	ED visits, hospital and ICU admissions	Administrative/clinical data ≥ 12 months	Captures system-level impact
Treatment Adherence	Compliance with prescribed therapy	Electronic monitoring, pharmacy refills	Modifiable determinant of outcomes
HRQoL	Impact on patient and family quality of life	Validated tools (PedsQL, CFQ-R, PAQLQ)	Patient-centered outcome
School Absenteeism	Days missed from school	Caregiver or school report	Proxy for disease burden
Caregiver Burden	Psychosocial and practical impact	Caregiver Strain Index, Family Impact Module	Family-centered assessment

COS—Core Outcome Set; ED—Emergency Department; ICU—Intensive Care Unit; FEV_1_—Forced Expiratory Volume in one second; PEFR—Peak Expiratory Flow Rate; HRQoL—Health-Related Quality of Life; PedsQL—Pediatric Quality of Life Inventory; CFQ-R—Cystic Fibrosis Questionnaire–Revised; PAQLQ—Pediatric Asthma Quality of Life Questionnaire.

**Table 2:** Key Barriers to Telemedicine Adoption and Corresponding Strategic Solutions.

Barrier	Impact	Proposed Strategy
Outcome heterogeneity	Limits evidence synthesis	Adoption of COS
Regulatory fragmentation	Variable quality	MDR/CE marking, FDA SaMD alignment
Limited interoperability	Workflow disruption	Mandatory EHR integration
Digital divide	Health inequities	Broadband and device investment
Lack of reimbursement	Unsustainable adoption	HTA-aligned reimbursement
Workforce burden	Clinician resistance	Training and workflow integration
Ethical concerns	Privacy and autonomy risks	Pediatric ethical frameworks

COS—Core Outcome Set; MDR—Medical Device Regulation (European Union); CE—Conformité Européenne (European regulatory marking); FDA—Food and Drug Administration (United States); SaMD—Software as a Medical Device; EHR—Electronic Health Record; HTA—Health Technology Assessment.

**Table 3:** Stratification of Telemedicine Tools According to Pediatric Respiratory Conditions Suitable for Online Monitoring.

Disease/Clinical Condition	Suitable Telemedicine Tools	Clinical Objectives/Rationale
Asthma	Symptom-tracking applications; digital asthma action plans; smart inhalers; remote spirometry (PEFR/FEV_1_ where feasible); teleconsultations	Supports early detection of loss of control, improves adherence, facilitates self-management, and reduces exacerbation-related visits
Cystic Fibrosis)	Home spirometry; remote physiologic monitoring; tele-rehabilitation/exercise platforms; adherence-monitoring tools; video consultations	Enables longitudinal lung function monitoring, promotes physical activity, supports treatment adherence, and reduces travel burden
Non–Cystic Fibrosis Bronchiectasis	Symptom-reporting platforms; adherence-monitoring systems; tele-rehabilitation; teleconsultations	Facilitates early recognition of infective exacerbations, supports airway clearance strategies, and improves continuity of care
Chronic Respiratory Failure	Home pulse oximetry; remote oxygen therapy monitoring; ventilator telemonitoring; video-assisted follow-up	Supports early detection of clinical deterioration, optimizes device management, and reduces unplanned hospital utilization
Neuromuscular Disorders with Respiratory Involvement	Ventilator parameter monitoring; symptom-tracking tools; caregiver-report platforms; teleconsultations	Enables proactive management of respiratory decline, enhances caregiver support, and improves care coordination
Sleep-Disordered Breathing/PAP Therapy	PAP adherence monitoring; device telemonitoring; teleconsultations	Evaluates treatment adherence and effectiveness, supports troubleshooting, and reduces unnecessary clinic visits
Technology-Dependent Children	Integrated monitoring dashboards; video consultations; caregiver support platforms	Enhances multidisciplinary coordination, improves safety surveillance, and supports caregiver confidence

FEV_1_—Forced Expiratory Volume in one second; PAP—Positive Airway Pressure; PEFR—Peak Expiratory Flow Rate.

**Table 4:** Advantages and Disadvantages of Online Consultations in Pediatric Chronic Respiratory Care.

Dimension	Advantages	Disadvantages/Risks
Access to Care	Improves access for families in remote or underserved areas; reduces geographic barriers	Dependent on internet connectivity and device availability; may exacerbate the digital divide
Continuity of Care	Enables more frequent follow-up and monitoring between in-person visits	Risk of fragmented care if not integrated with standard clinical pathways
Clinical Management	Facilitates early identification of symptom deterioration; supports proactive interventions	Limited physical examination; potential for missed clinical signs
Patient and Caregiver Burden	Reduces travel time, school absenteeism, and caregiver work disruption	Potential digital fatigue; increased perceived monitoring burden
Treatment Adherence	Allows use of digital reminders and electronic monitoring tools	Over-reliance on self-reported or variable-quality home data
Healthcare Utilization	May reduce unnecessary emergency visits and hospitalizations	Risk of false reassurance or delayed escalation to in-person care
Workforce Impact	Can optimize scheduling flexibility and multidisciplinary collaboration	Increased clinician workload, alert fatigue, and data management demands
Economic Considerations	Potential long-term cost savings through reduced acute care utilization	Upfront costs for platforms, devices, integration, and training
Psychosocial Effects	Enhances convenience and family-centered communication	Possible reduction in therapeutic relationship quality for some patients
Equity and Inclusion	Can broaden reach when supported by public infrastructure	Unequal adoption across socioeconomic, cultural, and literacy groups

**Table 5:** Research Priorities Beyond the Core Outcome Set.

Research Domain	Key Questions	Recommended Study Designs
Longitudinal effectiveness	Does telemedicine modify disease trajectory?	Long-term cohort studies
Mechanisms of action	Which components drive benefit?	Factorial designs
Behavioral science	How to improve adherence?	Adaptive behavioral trials
Implementation science	What enables sustainable adoption?	Hybrid effectiveness–implementation
Economic evaluation	Is telemedicine cost-effective?	Cost-effectiveness analyses
Equity research	Do effects differ by SES or region?	Stratified mixed-methods studies
System integration	How to embed telemedicine into care?	Comparative health systems research

SES—Socioeconomic Status.

## Data Availability

All the available data are included in the manuscript.
